# Healthcare Systems and Variation in Healthcare Utilization at the End of Life Across Countries

**DOI:** 10.1093/geroni/igaa057.1842

**Published:** 2020-12-16

**Authors:** Jennifer Ailshire, Cristian Herrar, Margarita Maria Osuna

**Affiliations:** 1 University of Southern California, Los Angeles, California, United States; 2 Organisation for Economic Cooperation and Development OECD, Paris, Ile-de-France, France; 3 University of Southern California Leonard Davis School of Gerontology, Los Angeles, California, United States

## Abstract

With rapid population ageing, providing better end-of-life care (EOLC) is becoming a source of social demand and financial pressure for public and private budgets in many countries. This paper uses data from harmonized end-of-life interviews in the HRS family of studies to assesses variation in health care utilization across different income groups and how they differ across different health care systems. Hospital stay did not vary across health care systems, but nursing home stays were lower in countries with either national or statist social health insurance systems. Hospice use was low in all countries, but particularly in national and social health insurance systems. Lower income was associated with greater use of nursing homes in both the private and social health care systems. Low income was also associated with greater use of hospice in national health service, but lower use in social health service.

